# Knowledge of reproductive rights and associated factors among Oda Bultum University students, eastern Ethiopia

**DOI:** 10.3389/frph.2024.1464352

**Published:** 2024-10-10

**Authors:** Amedin Mohammed Hussen, Ahmedin Aliyi Usso, Gebi Agero, Tewodros Desalegn, Hassen Abdi Adem, Mohammed Yuya, Addis Eyeberu, Adnan Abrahim Sani

**Affiliations:** ^1^ Public Health Expert, West Hararghe Health Office, Oromia Health Bureau, Addis Ababa, Ethiopia; ^2^ School of Nursing and Midwifery, College of Health and Medical Sciences, Haramaya University, Harar, Ethiopia; ^3^ Department of Public Health, College of Health Sciences, Arsi University, Asella, Ethiopia; ^4^ School of Public Health, College of Health and Medical Sciences, Haramaya University, Harar, Ethiopia

**Keywords:** knowledge, reproductive health, reproductive rights, university students, Ethiopia

## Abstract

**Background:**

Reproductive rights are an essential element of public health interventions to reduce adolescent and youth mortality and morbidity. A lack of knowledge about sexual and reproductive health is an important barrier that contributes to a variety of health and social issues. This study assessed the knowledge of reproductive rights among Oda Bultum University students, eastern Ethiopia.

**Methods:**

An institution-based cross-sectional study was carried out among 727 students from December 1 to 30, 2020. Participants were selected using a multistage sampling technique. Data were collected using a self-administered, pre-tested, and structured questionnaire. Data were entered into EpiData version 4.1 and analyzed using SPSS version 27. The study employed both bivariable and multivariable logistic regression analysis to determine the variables associated with knowledge regarding reproductive rights. The significance and degree of strength were declared at a *p*-value < 0.05 using an adjusted odds ratio with a 95% confidence interval.

**Results:**

The overall knowledge of reproductive rights among university students was 47.2% (95% CI: 43.3%, 50.9%). Male gender (AOR = 2.11, 95% CI: 1.50, 2.97), urban residence (AOR = 1.62, 95% CI: 1.16, 2.28), formal maternal education (AOR = 2.26, 95% CI: 1.62, 3.17), participation in a sexual and reproductive health club (AOR = 2.67, 95% CI: 1.74, 4.10), utilization of sexual and reproductive health services (AOR = 6.29, 95% CI: 4.22, 9.36), and discussion about sexual and reproductive health issues (AOR = 1.60, 95% CI: 1.11, 2.30) were the factors that improved the knowledge of reproductive rights.

**Conclusions:**

Almost half of the university students know about reproductive rights. Various factors identified were associated with the knowledge of reproductive rights among university students, including gender, residence, parental education level, engagement in sexual and reproductive health clubs, utilization of sexual and reproductive health services, and discussions about sexual and reproductive issues. Healthcare professionals at all levels should concentrate on offering excellent services related to reproductive health and establishing programs for specific education and counseling on reproductive rights for all well-behaved students.

## Introduction

Reproductive rights are the fundamental rights of all individuals and couples, irrespective of age or gender, to reproductive and sexual health, including the ability to determine the number, timing, and spacing of children (1). Human rights declarations protected sexual and reproductive rights (2). Concerns about sexual and reproductive health, which are essential to population and development initiatives, disproportionately affect young people. For young people's well-being, it is crucial to safeguard and advance their reproductive rights and provide them with the knowledge necessary to make well-informed choices (3, 4).

Youths, defined as people between the ages of 15 and 24, account for around 18% of the global population, the great majority of whom reside in developing nations. A time of biological and psychological transition from childhood to maturity, it is marked by increased sexual activity and the ability to reproduce sexually (1, 5). Access to services and information that support these decisions and advance the rights to sexual and reproductive health is part of ensuring reproductive rights. Insufficient knowledge about reproductive rights can result in several harmful health consequences for youths as they grow more mature, including unsafe sexual behavior, unwanted pregnancies, violence, and unprotected sex. These consequences account for 17% of the world's overall disease burden (6). The second highest cause of disease and death in developing countries is unprotected sex (4, 5, 7). Although young people and their parents in Sub-Saharan Africa have little knowledge of reproductive rights, the early onset of sexual relations and the high incidence of sexually transmitted diseases, particularly HIV/AIDS, lead to a lack of protection (8). In Ethiopia, teenage marriages and teenage pregnancies are prevalent. According to the 2016 Ethiopian Demographic Health Survey (EDHS), teenage girls give birth to 16% of all babies in Ethiopia, and 17% of these pregnancies are undesired or unplanned (9).

Even though sexual and reproductive health is vital for everyone, regardless of age, many people, especially youth in developing countries like Ethiopia, lack access to sexual and reproductive health care (10). Due to misunderstanding and insufficient awareness, many sexually active young people feel that they are unable to control or talk about certain elements of their sexual behavior (11). Research indicates that youth usually do not know how much their sexual rights are being violated or where to get social or legal assistance (12). In addition, poor quality rights-based interventions for sexual and reproductive health continue to be a serious problem in low- and middle-income countries (LMICs), such as Ethiopia (13). Research indicates that university students in Ethiopia know as little as 16.4%–57.7% about sexual and reproductive health rights (14–17). Ethiopia has ratified several international agreements concerning sexual and reproductive rights to address these problems, and several organizations have been focusing on sexual and reproductive health services and related rights in Ethiopia (8). However, the continued existence of these problems might be attributed, in part, to higher education institutions’ lack of national standards curriculam on sexual and reproductive rights for non-health science majors (8).

The youth's capacity to deal with violations was restricted by several issues, such as feelings of shame, guilt, and humiliation; fears of rejection by friends and family; concerns about confidentiality; and fears of not being believed or of being subjected to discrimination and cultural stigma (7). Another major obstacle that might result in many health risks and social issues is a lack of knowledge about sexual health (18).

There is little information available on reproductive rights and related issues in Ethiopia, especially in the overall setting of the study, despite data on the use of reproductive health care (19–21). In low- and middle-income countries, including Ethiopia, addressing the needs and issues related to youth's sexual and reproductive health is a top priority in population and development programs. Therefore, this study aimed to assess knowledge of reproductive rights and associated factors among students in Oda Bultum University, eastern Ethiopia.

## Methods and materials

### Study design and area

An institution-based cross-sectional study was conducted at Oda Bultum University from December 1 to 30, 2020. Oda Bultum University established in 2016, located in the West Hararghe Zone, 326 kilometers east of Addis Ababa, Ethiopia's capital. The university accepted 2,876 undergraduate students in undergraduate programs during the study period. Of these students, 1,770 were eligible for the study. The institution has one youth club that offers services related to HIV/AIDS and reproductive health and one clinic that delivers basic health care to students, faculty, and the local community.

### Population

All undergraduate students at the Oda Bultum University were the source population. Randomly selected undergraduate students at Odabultum University during the study period constituted the study population. The study included regular undergraduates who were 18 years old and above, while critically ill participants who could not respond and were absent from regular class sessions were excluded.

### Sample size and sampling procedure

The sample size was determined by Epi Info version 7.1, using a single population proportion formula with the following assumptions: a 95% confidence level, assuming a 31.6% proportion of reproductive health knowledge, a 5% margin of error, a design effect of 2 and a 10% non-response rate. Accordingly, a minimum of 730 students were required to participate in this study.

A multistage stratified sampling procedure was applied to determine study participants. First, Oda Bultum University is stratified by college (College of Agriculture, College of Agroindustry, Natural and Computational Sciences, Business and Economics, Natural Resources and Environmental Sciences, College of Humanity and Social Sciences, and Engineering); then, the sample size proportionally allocated to each college. Second, the assigned samples to each college were proportionally allocated to their respective departments and programs/units found under each college, along with their years of study, using the actual number of learning undergraduate students reviewed from the most recent student lists or registrations available in the Associate Registrar Office of Oda Bultum University during the study period, 2020. We created a sampling frame for each college using student registration numbers and then selected participants using a systematic random selection technique.

### Data collection tool and procedure

Data were collected from participants using a structured self-adminstered questionnaire after the classroom was arranged. The questionnaire was adapted from the available published literatures (8, 9, 17, 20–22). The questionnaire contained information on socio-demographic factors (age, gender, ethnicity, religion, marital status, address, family size, paternal and maternal education, paternal and maternal main occupation, and the family's wealth index), school-related factors (type of school attended in lower education, college of the study, and year of the studies), reproductive factors (history of sexual experience, age and time at first sex, number of sexual partners, hearing about sexual and reproductive health information and their primary source of information, participation in a reproductive health club, discussion about sexual and reproductive health issues, advice on sexual and reproductive health issues, and use of sexual and reproductive health services) and knowledge of reproductive rights.

#### Knowledge of reproductive rights

It was measured using 24 dichotomous (yes/no) items of reproductive rights knowledge. The response of each item was coded ′1′ when the participant responded ‘yes’ to the correct questions and ‘0’ when the participant responded ‘no’ to the correct question. Then, the composite index score was computed from 24 dichotomous items, and the total score ranged from 0 to 24. The internal consistency of the items was confirmed, with acceptable consistency (Cronbach *α* = 0.83). The participants who scored the mean and above were considered to have good knowledge of reproductive rights and had poor otherwise (8).

#### Wealth index

Was evaluated using a standard instrument that included 38 dichotomous (yes/no) questions about three aspects of the family's wealth level (domestic animals, durable assets, other productive assets, and housing circumstances) (9). We found high internal consistency among questions (Cronbach *α* = 0.81) and used principal component analysis using the varimax rotation approach to determine composite wealth index variables and participant wealth status.

### Data quality control

The questionnaires were adapted from standard instruments and related published literature to maintain the quality of the data. We pretested the tools on 5% of the total sample at Dire Dawa University in Ethiopia to check the validity of the tools. After the pretest, ambiguous words were simplified based on recommendations from participants. Before actual data collection, data collectors and supervisors were trained for one day on the objective of the study and data collection techniques. Ten health professionals facilitated the overall data collection, and two public health officers supervised data collection with the principal investigator. The study participants filled out the questionnaire in the lecture halls at the same time. The participant's privacy was not compromised by the other participants. Supervisors and principal investigators strictly supervised and validated the collected data.

### Data processing and analysis

The data were entered into EpiData version 3.1 and analyzed with SPSS version 27 after being checked for correctness and completeness. Descriptive statistics such as frequencies, measures of central tendency, and measures of dispersion were employed to characterize the individuals. Before any analysis, the internal consistency of the items used to measure each composite index was checked by reliability analysis (using Cronbach α). We found acceptable reliability tests for knowledge of reproductive right items (Cronbach α = 0.83) and wealth index items (Cronbach α = 0.81). The composite wealth index score and wealth status of the students’ households were determined using Principal Component Analysis and the varimax rotation method. Predictors with a *P*-value of <0.25 in bivariable analyses be included in the multivariable. The factors independently associated with knowledge about reproductive rights were identified using multivariable logistic regression analyses. The adjusted odds ratio (AOR) (95% CI) was used to report the strengthening of the association, with overall significance noted at a *P*-value <0.05.

## Results

### Socio-demographic characteristics

A total of 727 students participated in this study, with a response rate of 99.6%. Four hundred forty (60.5%) participants were male. The mean (±SD) age of the participants was 22.71 ± 2.25 years. The majority (77.3%) of participants were in the 21–24-year age group. More than half (56.4%) of the students were from rural areas. A majority (46.4%) of the participants had mothers who had no formal education; half (50.9%) of paternals’ occupations were Farmar, about 33.3% of students were from Poor families, followed by 33.4% from Medium and 33.3% from Rich household families. The majority (80.2%) of students attended their preparatory education at governmental schools (Table 1).

**Table 1:** Socio-demographic characteristics of students in Oda Bultum University, eastern Ethiopia, 2021 (*n* = 727).

Characteristics		Frequency	Percent (%)
Sex	Male	440	60.5
	Female	287	39.5
Age (in years)	18–20	30	4.1
	21–24	562	77.3
	≥25	135	18.6
Residence area	Urban	317	43.6
	Rural	410	56.4
Marital status	Single	674	92.7
	Married	40	5.5
	Divorced	13	1.8
Religion	Muslim	247	34.0
	Orthodox	274	37.7
	Protestant	206	28.3
Ethnicity	Oromo	464	63.8
	Amhara	149	20.5
	Tigre	95	13.1
	Others^a^	19	2.6
Family size	≤5	335	46.1
	>5	392	53.9
Maternal education	No formal education	337	46.4
	Had formal education	390	53.6
Paternal education	No formal education	209	28.7
	Had formal education	518	71.3
Maternal main occupation	Housewife	411	56.5
	Government employee	143	19.7
	Merchant	141	19.4
	Others^b^	32	4.4
Paternal main occupation	Farmer	370	50.9
	Government employee	236	32.5
	Merchant	99	13.6
	Others^c^	22	3.0
Preparatory school types	Government	583	80.2
	Private	144	19.8
Faculty	Social sciences	229	28.7
	Natural sciences	498	71.3
Year of the study	2nd year	463	63.7
	3rd year	228	31.4
	≥4th year	36	5.0
Wealth index	Poor	242	33.3
	Medium	243	33.4
	Rich	242	33.3

^a^ Somale/Sidama/Gurage/wolayta.

^b^ Daily labor.

^c^ Daily laborer.

### Reproductive characteristics

The majority (78.7%) of participants had sexual experience. Of those who had sexual experience, 55.5% had multiple sexual partners in their lives. Around 71.7% of students were participated on sexual and reproductive clubs, and 32.5% of students were utilized reproductive health services. In addition, 40.7% of students were discussed about reproductive health issues with any body (Table 2).

**Table 2:** Reproductive characteristics of students in Oda Bultum University, eastern Ethiopia, 2021 (*n* = 727).

Characteristics		Frequency	Percent (%)
History of sexual intercourse	Yes	155	21.3
	No	572	78.7
Age at first sexual intercourse (*n* = 155)	<15 years	64	41.3
	15–19 years	70	45.2
	20–24 years	21	13.5
Number of sexual partner (*n* = 155)	One	69	44.5
	Two	53	34.2
	Three and above	23	21.3
Heard about SRH issues	Yes	388	53.4
	No	339	46.6
Where did you heard about SRH issues first (*n* = 338)?	Parents	80	23.7
	Peers	49	14.5
	School teacher	73	21.6
	Social media	79	23.4
	Health worker	40	11.8
	Media (TV/Radio)	64	18.9
	Other^a^	28	8.3
Participation on SRH club	Yes	521	71.7
	No	206	28.3
Utilization of SRH services	Yes	236	32.5
	No	491	67.5
Type of SRH services used (*n* = 236)	Condom	62	38.8
	ABC education	62	38.8
	STI and HIV education	31	19.4
	Other^b^	5	3.6
Discussing about SRH issues	Yes	296	40.7
	No	431	59.3
With whom did you discuss about SRH issue (*n* = 296)	My family members	117	39.5
	My friends	87	29.4
	Health worker	62	20.9
	My school teacher	18	6.1
	Other^c^	12	4.1

^a^ RH clubs, student and boyfriend.

^b^ Contraceptives, abortion care.

^c^ Religious leader and grandmother's in law; SRH, sexual and reproductive health.

### Knowledge of reproductive rights

The mean (±SD) score for knowledge about reproductive rights is 14.36 ± 3.65. Three hundred forty-three (47.2%) students at the university had a good knowledge of reproductive rights (95% CI: 43.3%, 50.9%).

The majority (61.8%) of participants agreed that families had the right to choose circumcision for their female children. Nearly half (48.1%) agreed that married women should be able to choose how many children they want without the approval of their spouse or partner. Half of the participants (50.2%) agreed that women had the right to decline sexual activity without their boyfriend's consent. Approximately 46.6% of participants believed that boys should be allowed to engage in sexual activity without their partner's consent. More than half of the participants (55.7%) agreed that females have the freedom to make sexual and reproductive health decisions without their parents’ permission. The majority of them (45.3%) agreed that unmarried couples have the right to use contraception other than condoms (Table 3).

**Table 3:** Responses to items of knowledge of reproductive rights among Oda bultum university students, eastern Ethiopia, 2021 (*n* = 727).

Items	Yes (%)	No (%)
Do families have right to decide about their female child to be circumcised?	449 (61.8)	278 (38.2)
Do girls have the right to dismiss her arranged marriage without their family's consent?	360 (49.5)	367 (50.5)
Do youths have the right to mate selection without their family's consent?	427 (58.7)	300 (41.3)
Can a married woman say no to have children if she doesn't want?	350 (48.1)	377 (51.9)
Do women have the right to say no to have sex without their husband's/partners desire/need?	365 (50.2)	362 (49.8)
Do youths have the right that their use of reproductive health services is to be kept confidential?	418 (57.7)	309 (42.5)
A man shouldn't have sex whenever he wants irrespective of his girlfriend's wish?	388 (53.4)	339 (46.6)
Do youths have the right to ask each other for HIV testing before sexual engagements?	433 (59.6)	294 (40.4)
Do youths have the right to have information on reproductive health facilities	483 (66.4)	244 (33.6)
Do youths have rights to access information of reproductive rights at higher institutions?	464 (63.8)	263 (36.2)
Do you think that youths have right to be free from all forms of discrimination due to their reproductive and sexual orientation?	381 (52.4)	346 (47.6)
Do boys have no special right to be protected from sexual exploitation and abuse	357 (49.1)	370 (50.9)
Do all girls have the right to access to autonomous reproductive choices including choices relating to safe abortion?	328 (45.1)	399 (54.9)
Do girls have the right to resist genital mutilation against their families will?	402 (55.3)	325 (44.7)
Do youths have full right to access reproductive health service without parents consent	455 (62.6)	272 (37.4)
Do youths have the right to decide on sexual and reproductive health issues of themselves without their parents’ consent?	405 (55.7)	322 (44.3)
Do all women have right to autonomous reproductive choices to use contraceptive	364 (50.1)	363 (49.9)
Do girls have right to autonomous reproductive choice without partner's consent	328 (45.1)	399 (54.9)
Do students have a right to freedom of assembly and political participation to influence governments to place *a priori*ty on sexual and reproductive health?	422 (58.0)	305 (42.0)
Do students have the right to access new reproductive technologies	411 (56.5)	316 (43.5)
Do you think that all students must be free to enjoy and control their sexual and reproductive life?	381 (52.4)	346 (47.6)
Do youths have the right to form an association or clubs that aims to promote their sexual and reproductive health?	380 (52.3)	347 (47.7)
Do unmarried woman have the right to maternity leave with adequate social security benefits?	377 (51.9)	350 (48.1)
Do unmarried couples have the right to use contraceptives other than condoms?	329(45.3)	398(54.7)

### Factors associated with knowledge of reproductive rights

In the bivariable analysis, students’ gender, resident area, age, religion, marital status, maternal educational status, family wealth quantile, participation in sexual and reproductive health (SRH) clubs, use of SRH services, discussion about SRH issues, school type, faculty, and year of study were all associated with knowledge of reproductive rights. However, in multivariable analysis, students’ gender, living place of residence, maternal education, involvement in an SRH club, and utilization of SRH were the independent characteristics associated with knowledge concerning reproductive rights among university students (Table 4).

**Table 4:** Factors associated with knowledge of reproductive rights among Oda Bultum University students, eastern Ethiopia, 2021 (*n* = 727).

Variables		Knowledge of reproductive rights		COR (95%CI)	AOR (95%CI)
		Good (%)	Poor (%)		
Gender	Female	108 (37.6)	179 (62.4)	1	1
	Male	235 (53.4)	205 (46.6)	1.90 (1.40, 2.57)***	2.11 (1.50, 2.97)***
Age (in years)	18–20	11 (36.7)	19 (63.3)	1	1
	21–24	259 (46.1)	303 (53.9)	1.48 (0.69,3.16)	1.58 (0.65, 3.83)
	≥25	73 (54.1)	62 (45.9)	2.03 (0.90,4.60)	2.16 (0.84, 5.59)
Residence area	Urban	167 (52.7)	150 (47.3)	1.48 (1.10, 1.99)**	1.62 (1.16, 2.28)**
	Rural	176 (42.9)	234 (57.1)	1	1
Religion	Muslim	110 (44.5)	137 (55.5)	1	1
	Orthodox	130 (47.4)	144 (52.6)	1.24 (0.80, 1.59)	1.48 (0.99, 2.22)
	Protestant	103 (50.0)	103 (50.0)	1.24 (0.86, 1.80)	1.37 (0.89, 2.11)
Preparatory School type	Private	77 (53.5)	67 (46.5)	1.37 (0.95, 1.97)	1.04 (0.67, 1.61)
	Government	266 (45.6)	317 (54.4)	1	1
Year of the study	2nd year	207 (44.7)	256 (55.3)	1	1
	3rd year	115 (50.4)	113 (49.6)	1.37 (0.87, 3.44)	1.15 (0.80, 1.66)
	≥4th year	21 (58.3)	15 (41.7)	1.62 (0.92,1.74)	1.49 (0.67, 3.28)
Maternal education	No formal education	129 (38.3)	208 (61.7)	1	1
	Had formal education	214 (54.9)	176 (45.6)	1.96 (1.46, 2.64)***	2.26 (1.62, 3.17)***
Family wealth index	Poor	114 (47.1)	128 (52.9)	1	1
	Medium	108 (44.6)	134 (55.4)	0.91 (0.63,1.29)	0.90 (0.60, 1.35)
	Rich	121 (49.8)	122 (50.2)	1.11 (0.78, 1.59)	1.24 (0.82, 1.86)
Participated on SRH club	Yes	244 (46.8)	277 (53.2)	**1.51 (1.10, 2.09)** *	2.67 (1.74, 4.10)***
	No	99 (48.1)	107 (51.9)	1	1
Used SRH services	Yes	171 (72.5)	65 (27.5)	4.88 (3.47, 6.86)***	6.29 (4.22, 9.36)***
	No	172 (35.0)	319 (65.0)	1	1
Discussed about SRH issues	Yes	167 (56.4)	129 (43.6)	1.87(1.39, 2.53)***	**1.60(1.11, 2.30)** *
	No	176(40.8)	255(59.2)	1	1

* *p*-value < 0.05; **p-value < 0.01; ***p-value < 0.001; AOR, adjusted odds ratio; COR, crude odds ratio; SRH, sexual and reproductive health.

The meaning of the bold value is indicated for variable having *p*-value <0.05; while the non-bold value indicated for variable having *p*-value ≥0.05.

Male students were twice as likely as female students to know about reproductive rights (AOR = 2.11, 95% CI: 1.50–2.97). Students from urban areas had 1.6 times higher odds of knowing about reproductive rights compared to those from rural areas (AOR = 1.62, 95% CI: 1.16–2.28). Students whose mothers had a formal education were 2.26 times more likely to be aware of reproductive rights than those whose mothers had no formal education (AOR = 2.26, 95% CI: 1.62–3.17). Similarly, students who participated in the SRH club were 2.67 times more likely to know about reproductive rights than those who did not (AOR = 2.67, 95% CI: 1.74–4.10). Furthermore, students who had previously used SRH services were 6.29 times more likely to be aware of reproductive rights than those who had never used it (AOR = 6.29, 95% CI: 4.22–9.36). Finally, the students who discussed SRH issues with anybody were 1.6 times more likely to know about reproductive rights than those who had not discussed it yet (Table 4).

## Discussion

In this study, we assessed the level of knowledge on reproductive rights and associated factors among Oda Bultum University students. Less than half (47.2%) of the students understood their reproductive rights. Male students, living in urban areas, having a mother's formal education, involvement in SRH club, use of SRH services, and discussion of SRH concerns with others were independent predictors of reproductive rights knowledge among University students.

This study indicated that about 47.2% of students were knowledgeable about reproductive rights. This finding was similar with the finding of the studies conducted in Mizan Tepi, southern Ethiopia (46.6%) (23), and Shire, northern Ethiopia (47.1%) (17). However, this finding was higher than the study conducted in Aksum, Northern Ethiopia (16.4) (14). On the other hand, the finding of this study was lower than the studies conducted in Haramaya, Eastern Ethiopia (52.2%) (16), Wolaita Sodo, southern Ethiopia (54.5%) (8), southwest Nigeria (60.3%) (24), and Nepal (83.3%) (22). This disparity could be explained by differences in the research population and participants’ educational backgrounds. For example, the study done in Wolaita Sodo included health science students and participants from the health science college were more likely to be aware of reproductive rights than those from other colleges (8). In addition, methodological differences across research may account for this disparity. For instance, a study conducted in Nigeria used fewer items to assess the level of knowledge regarding reproductive rights and used a mixed-methods methodology (24). Furthermore, this variation could be attributed to the difference in study settings and populations among studies.

This study revealed that male students were more likely to know about reproductive rights than female students. This finding was supported by the study conducted in Aksum, northern Ethiopia (14). This difference may attributed to the presence of social and cultural barriers that inhibit females from engaging in discussions about sexual and reproductive health (25). In addition, the patriarchal systems, gender inequality, and the violation of women's rights, as well as the reluctance of females who participate in sexual and reproductive health education, may also contribute to this disparity (26).

Students from urban residents were more likely to be aware of reproductive rights than those from rural residents. This finding was consistent with studies carried out in Aksum, northern Ethiopia (14), Wolaita Sodo, southern Ethiopia (8) and Gondar, Northwest Ethiopia (15). This could be because students from urban areas have more access to reproductive health information. In addition, urban family units had higher levels of education and had more discussions with their children about sexual and reproductive health than rural ones (14). Developing strategies that help to increase the window access to information on sexual and reproductive health for rural residences is required to improve the knowledge of reproductive rights.

Maternal education is significantly associated with knowledge about reproductive rights. Students whose mothers had a formal education were more likely to be aware of reproductive rights than students whose mothers had not. This finding, supported by studies carried out in Haramaya, eastern Ethiopia (16), Adet, northwest Ethiopia (27) and Riyadh, Saud Arabia (18), implies that students from educated families discussed reproductive rights with their families. Educated families might not restrict their children from discussing sexual and reproductive rights, which may enhance the children's knowledge. Given that, encouraging family members to discuss reproductive health in the household is imperative to improve the knowledge level of reproductive rights in the community.

The students who participated in the sexual and reproductive health club were more likely to know about reproductive rights compared to those who did not participate. This finding has been strengthened by the studies conducted in Shire, northern Ethiopia (17) and Wolaita Sodo, southern Ethiopia (8). Participating in a sexual and reproductive health club allows individuals to discuss their ideas and feelings, concerns, difficulties, and problems with an expert, which helps them have a better understanding of their experiences and find an existing choice. Given that, providing routine and effective sexual and reproductive health counseling for youth age groups at any contact with health facilities is essential to improve the level of knowledge of reproductive rights.

In addition, the study found that the use of reproductive health care services is substantially associated with knowledge about reproductive rights. Students who used reproductive health services were six times more likely to understand reproductive rights than their counterparts. A cross-sectional study conducted in southern Ethiopia provided support for this finding. These could be attributed to a higher likelihood of receiving advice from healthcare providers during service utilization and a greater emphasis on understanding what they use (8). This means that the availability of reproductive health services and intervention-based sexual and reproductive health education at the university is essential for increasing students’ knowledge of reproductive rights.

Furthermore, students who discussed sexual and reproductive health issues with others were more likely to understand reproductive rights than their peers. This could be due to enhanced knowledge gained through discussion and experience sharing. These findings have been supported by studies conducted in Aksum, northern Ethiopia (14), Wolayta Sodo, southern Ethiopia (8), and Adet, northwest Ethiopia (27). A providing strategy-based health education about sexual and reproductive health for all adolescent groups is important to enhance the level of reproductive rights.

The study's strength was that it used a large sample size and multistage stratified sampling method and that we included individuals from across the region of the country. This study has some limitations even though it adheres closely to scientific procedures. For example, because the study had been limited to one public university in Ethiopia, it could not represent all governmental and private universities in the country. In addition, because sexual and reproductive issues are sensitive, the findings of the study might influenced by the social desirability bias. Furthermore, it is difficult to determine the temporal correlations between variables because the study was cross-sectional.

## Conclusions

According to this study, 47.2% of Oda Bultum University students were know of their reproductive rights. Male gender, urban residence, higher maternal education level, participation in SRH clubs, use of SRH services, and discussion of SRH concerns with others were independent predictors of reproductive rights awareness among university students. Public health facilities at all levels should work with existing higher education institutions to encourage students to participate in reproductive health concerns. In addition, healthcare service providers at all levels should prioritize delivering high-quality reproductive health services, as well as establishing special education and counseling programs on reproductive rights for all disciplinary students. Overall, the Ethiopian Ministry of Education and the Ministry of Health should coordinate to design strategy-based curricula that incorporate reproductive health and rights in high schools and institutions of higher learning, which is essential to enhance the knowledge level of reproductive rights.

## Data Availability

The original contributions presented in the study are included in the article/Supplementary Material, further inquiries can be directed to the corresponding author.
